# What Is the Biological Function of Uric Acid? An Antioxidant for Neural Protection or a Biomarker for Cell Death

**DOI:** 10.1155/2019/4081962

**Published:** 2019-01-10

**Authors:** Dequan Liu, Yu Yun, Dechun Yang, Xinyu Hu, Xianxiang Dong, Nan Zhang, Lumei Zhang, Hua Yin, Weigang Duan

**Affiliations:** ^1^The Third Affiliated Hospital and School of Basic Medicine, Kunming Medical University, Kunming 650500, China; ^2^Key Laboratory of Molecular Biology for Sinomedicine, Yunnan University of Traditional Chinese Medicine, Kunming 650500, China

## Abstract

The main aim of the present study was to investigate the biological function of uric acid. The level of uric acid in different organs in normal male rats was determined with uric acid assay kits, and the expression level of genes in the organs was determined by RNA quantitative sequencing. The correlation analysis between uric acid in the organs and gene expression (measured by FPKM value) was made. Serum uric acid (SUA) in patients with breast cancer or with breast benign tumor was assayed when the diagnosis was made, and SUA in patients with breast cancer was also assayed just after chemotherapy. There were 1937 mRNAs whose expression level significantly correlated with the level of uric acid, and most of which were associated with purine or nucleoside metabolism, cellular metabolism, cell cycles, and cell death pathways. Further analysis showed that the level of uric acid was highly correlated with cell death rather than cell viability. The level of SUA in patients with breast cancer was higher than that in patients with breast benign tumor, and the SUA increased after chemotherapy. All the results suggested that uric acid was mainly synthesized from local nucleosides degraded from dead cells, and uric acid could be an important biomarker for cell death rather than an antioxidant for neural protection.

## 1. Introduction

Hyperuricemia is an old topic in the field of metabolic disorders and a common fundamental disease for gout, renal dysfunction, and other cardiovascular diseases [1]. The disease is attracting more and more eyes of biologists and medical scientists because of the high morbidity and economic burden [2]. Hyperuricemia has a male dominance and can be diagnosed by the level of serum uric acid (SUA) above 420 *μ*M (70 *μ*g/ml) [2].

The direction cause of hyperuricemia is the accumulation of uric acid in the body. Uric acid is the final product of purine nucleoside metabolism, synthesized by xanthine dehydrogenase (Xdh) in humans though it can be further transformed into allantoin by uricase (Uox) in other animals except some birds and reptiles [3]. Uric acid can be found in cells, tissues, and organs, and the level of uric acid is different from organ to organ. It was supposed that the liver was the most important organ to generate uric acid [4, 5], but strangely, not the organ with the highest level of uric acid [6]. The dominant source of uric acid (about 2/3 or more) is generated from endogenous purines, and the rest from the exogenous [4]. It is certain that two-thirds or more uric acid is excreted through the kidney, and the rest through feces [4]. Although there were systematic data about the distribution of uric acid in rats [6], the significance of uric acid in different organs was poorly understood.

It was believed that uric acid was a metabolic waste of nucleosides just like urea for proteins, since there was almost no functional disturbance if the level of serum uric acid was lowered deeply by Uox, say rasburicase [7]. However, some reports declared that uric acid played roles in some physiologic functions and should not be eliminated thoroughly [8, 9]. Due to its antioxidant activity, uric acid was thought to protect neuronal cells, consequently facilitating brain evolution or development [8, 10, 11], and also play a role in maintaining the blood pressure [8]. However, the antioxidant activity of uric acid is not powerful than either hydrophilic vitamin C or hydrophobic vitamin E according to its chemical structure. The effects resulted from the antioxidant activity can be easily substituted by intake of the two vitamins [12, 13] and other foods containing reducing chemicals [14]. Therefore, its antioxidant activity is not as important as previously supposed. So, the function of uric acid in the organs is still unclear. In the present study, uric acid in different rat organs and their mRNA expression level would be determined, and the relationship between them would be analyzed to find its function.

## 2. Materials and Methods

### 2.1. Materials

Male Sprague-Dawley (SD) rats aged 2 months and weighing 180-220 g were obtained from Kunming Medical University, Kunming, China. Rats were housed at 22°C temperature, at 45-55% humidity-controlled conditions, and under natural light. Clinical data were collected from the Third Affiliated Hospital, Kunming Medical University. This project was approved by the Experimental Animal Committee of Kunming Medical University and the Medical Ethics Committee of Kunming Medical University.

Uric acid was purchased from Tokyo Into Industrial Co. Ltd. (Tokyo, Japan). Uric acid assay kits of the phosphotungstic acid method and protein assay kits of the BCA (bicinchoninic acid) method were purchased from Nanjing Jiancheng Bioengineering Institute (Nanjing, China). TRIzol Plus RNA Purification Kit was purchased from Invitrogen (Carlsbad, CA, USA). Ultrapure water was obtained from the Milli-Q water purification system manufactured by the EMD Millipore Group (Darmstadt, Germany). The NanoDrop ND-1000 spectrophotometer was manufactured by PeqLab, Erlangen, Germany. The multimicroplate reader of Infinite 200 PRO was manufactured by Tecan Group (Männedorf, Switzerland). Other instruments or reagents used in the present study were made in China.

### 2.2. Animal Treatment and Uric Acid Assay

Animal treatment followed the methods of Yun et al. [6]. Briefly, SD rats were fasted for 36 h before sacrificing. The rats were intraperitoneally anaesthetized with urethane (1.0 g/kg). The abdomen of the rat was opened, blood samples were drawn via the abdominal aorta, and organs including the liver, spleen, lung, bladder, pancreas, kidney, testicle, brain, heart, ectogluteus, duodenum (5 cm), and the last 5 cm of the ileum were harvested. The intestinal tract was opened and the inner wall was cleaned with a cotton swab and was rinsed with 1 ml normal saline twice. The sample of the organs was frozen at -40°C for use or homogenized on ice immediately.

The concentration of uric acid (*μ*g/ml) in the serum samples and the supernatant of the tissue homogenate was assayed with uric acid assay kits according to the standard operation procedure (SOP) provided by the manufacturer. The protein in all the samples was assayed with protein assay kits.

### 2.3. mRNA Quantitative Sequencing

After the organs of the rats including the brain, kidney, lung, liver, heart, stomach, duodenum, and terminal ileum were harvested, about 200 mg tissue was sampled. The sample was frozen with liquid nitrogen and ground to powder. The total RNA in the powder was extracted and purified by TRIzol Plus RNA Purification Kit. RNA quantity and quality were measured by the NanoDrop ND-1000 spectrophotometer. RNA integrity was assessed by standard denaturing agarose gel electrophoresis [15, 16].

Double-stranded cDNA (ds-cDNA) was synthesized from the total RNA using an Invitrogen SuperScript ds-cDNA synthesis kit in the presence of 100 pmol/l oligo dT primers. The cDNA was sequenced by Sangon Biotech (Shanghai, China). The expected value of FPKM (fragments per kilobase of transcript sequence per million base pairs sequenced) was used for expression normalization [17, 18]. The relationship analysis of the FPKM value of a gene between different organs and the pathway analysis associated with related genes [15] were also made by Sangon Biotech.

### 2.4. Clinical Data

Clinical data including 203 cases of breast cancer (aged 24-80) and 100 cases of benign breast tumor (aged 28-61) were provided by the Third Affiliated Hospital, Kunming Medical University. All the cases were from female patients and confirmed by pathological diagnosis. Cases with severe hepatic and renal dysfunction or complicated with other tumors were excluded. SUA was assayed with uric acid assay kits of uricase methods, and the Ki67 antigen in cancer tissue was assayed with ELISA kits by the clinical laboratory of the hospital.

### 2.5. Statistical Analysis

Values were expressed as mean ± SD (standard deviation). Student's *t*-test was performed to compare means between different groups. Bivariate correlations of Pearson's method (two-tailed) were performed to find the relationship between the level of uric acid and the FPKM values of the organs. Statistical significance was accepted at *P* < 0.05.

## 3. Results

### 3.1. Distribution of Uric Acid in Different Organs in Normal Rats

**Figure 1:**
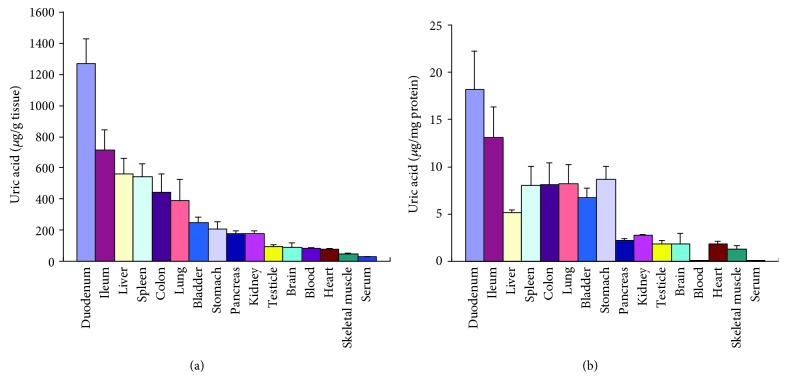
Distribution of uric acid in male rat's organs (mean + SD, *n* = 10, data cited from our previous study [6]). Ileum: the last 5 cm of the ileum; skeletal muscle: the ectogluteus.

The distribution of uric acid in different organs was showed in Figure 1. The organ with the highest level of uric acid was the duodenum, then the ileum and liver, belonging to the alimentary system [6] and suggested that the alimentary system was a dominant place for uric acid distribution.

**Figure 2:**
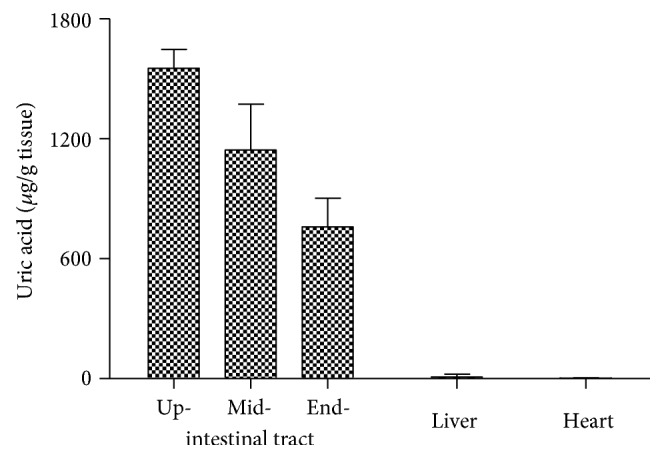
Distribution of uric acid in neonatal rats (mean + SD, *n* = 3).

In neonatal rats (less than 2 hours after birth), uric acid in the intestinal tract was similar to that in normal rats. However, uric acid in the heart and liver, unlike that in normal rats, was very low, different from that in normal rats (Figure 2).

### 3.2. Relationship between the Level of Uric Acid and Pathways in Different Organs

The results of mRNA quantitative sequencing showed that there were 32,662 mRNAs sequenced in every organ. The correlation between uric acid and the FPKM value was performed to find the relationship. Since mRNAs with multiple zero values of FPKM were unsuitable to perform correlation analysis, only 7604 mRNAs were taken into account, and 1937 mRNAs with a *P* value below 0.05 were selected. The pathways they could be involved in were also analyzed; 56 pathways with significance (*P* < 0.05) were screened out (Table S1), and the top 20 were showed in Table 1. Most pathways were associated with purine or nucleoside metabolism, cellular metabolism, cell cycles, and cell death.

### 3.3. Relationship between the Level of Uric Acid and Gene Expression in Different Organs

Uric acid is directly produced from xanthine by Xdh, while Ada (adenosine deaminase) is an important assistant to the kinase [19]. Their expression levels were both upregulated in organs where uric acid was high [6, 19]. However, as for Uox, there was no significant correlation between its expression level and uric acid (Table 2).

ATP is generated mainly by the Krebs cycle in the mitochondria and is a key fuel to drive cellular function. ATP is a polar molecule with negative charges, unlikely provided by neighbor cells or cells faraway. The increase of ATP will be associated with the upregulation of ATP synthase. However, there was no significant correlation between the level of uric acid and ATP synthase expression, though ATPases were upregulated in the brain and heart (both of Atpaf1 and Atpaf2, Table 3). On the other hand, Na^+^-K^+^-ATPase, a key enzyme concluding 8 subunits to maintain cellular polarization, is highly associated with cell viability; only one of the subunits was merely, but negatively, correlated with the level of uric acid (Table 3).

The main way of physiological cell death is regulated cell death (RCD); among which, caspase 3 (Casp3) is an executor to directly cause cell death [20, 21]. Of course, there was a good correlation between the level of uric acid and the enzyme (Table 3).

MKi67 is a cell cycle and tumor growth marker presenting only in the nuclei of cycling cells and upregulated in cycling cells [22, 23]. In Table 3, there was also a good correlation between the expression level of MKi67 and the level of uric acid, though the correlation coefficient (*R* = 0.7768) was smaller than that of Casp3 (*R* = 0.9045).

### 3.4. SUA in Breast Cancer

**Figure 3:**
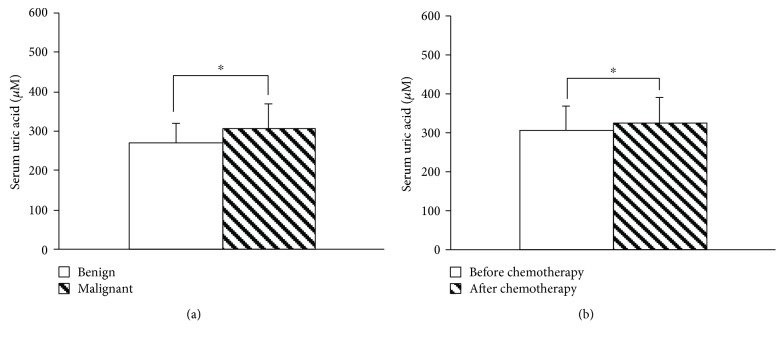
Serum uric acid (SUA) in patients with breast tumors (mean ± SD). The level of SUA before treatment in patients with malignant breast tumor (breast cancer) was higher than that in patients with benign breast tumor. (a) Benign group (*n* = 100) and malignant group (*n* = 203), *P* ≤ 0.001, independent samples *t*-test. When patients with breast cancer are treated with chemotherapeutics, the level of SUA further increased. (b) *n* = 203, *P* ≤ 0.001, paired *t*-test.

Figure 3(a) shows that the level of SUA in patients with breast cancer was higher than that in patients with benign breast tumor. Ki67 is a factor for tumor growth in human [22, 23]. However, there was no correlation between the level of SUA and Ki67 (Figure S1) in the patients with breast cancer. The patients that suffered with breast cancer were treated with chemotherapeutics, and when a treatment course was ended, the level of SUA increased to some extent (Figure 3(b)).

## 4. Discussion

The function of uric acid in the body was a hot topic. Some studies believe that uric acid might play some roles in physiological activities, like to protect neurons from oxidative damage and to maintain blood pressure [8, 24]. However, there was an opposite opinion that regarded the metabolite just as a waste [25, 26].

### 4.1. Uric Acid Synthesized from Local Degraded Nucleosides

In all the organs the present study involved, the expression level of Xdh was highly correlated with the level of uric acid (Table 1), and so was Ada, an enzyme indirectly associated with uric acid synthesis. The results suggested that uric acid was locally synthesized from the degraded nucleosides. As for the urate destroyer, Uox was highly expressed only in the liver (Table 1). The results suggested that uric acid was almost a final product of purines in the extrahepatic organs in rats and can be transported to the liver for further degradation.

### 4.2. Uric Acid Unlikely to Play an Important Role to Protect Neurons or Maintain Blood Pressure

Neurons predominantly distribute in the brain. According to the known understandings, the rat brain is about 0.4 percent of the body weight but consumes 10-20 percent of the cardiac output. Because the brain has to consume massive oxygen to maintain its complicated function, genes directly associated with ATP synthesis should be expressed at a high level. Unsurprisingly, the expression level of the genes was overall higher than that in other organs (Table 3). Theoretically, in the process of biological oxidation, many oxygen radicals will be also generated and then could damage neurons. Based on knowledge, some reports even deduced that uric acid played a role in neuroprotection [8] and Uox deficiency, a factor facilitating uric acid increase, was considered as a big step for human brain evolution and development [10, 11]. However, Uox deficiency is a common phenomenon in lower animals like birds and reptiles [3]. On the other hand, the heart is the second organ with a high level of energy metabolism. Unfortunately, the level of uric acid in the organ was also low (Figure 1). Similarly, the duodenum was the organ with the highest level of uric acid, but there were no reports that believed that the intestinal tract was an organ with high energy consumption. Therefore, the present study proved that uric acid was unlikely to play an important role in protecting neurons by quenching oxygen radicals, at least in a physiological state, though it is a real reductive substance.

Epidemic data suggested that hyperuricemia highly correlated with hypertension [27]. However, the relationship between uric acid and blood pressure was concomitant rather than causal. Both diseases are age-dependent, and there were rare concrete experimental evidences supporting the hypothesis that hyperuricemia could result in hypertension and vice versa. Further, there were almost no clues in uric acid-lowering drugs associated with blood pressure lowering [7]. From the analysis, uric acid was unlikely to play an important role in maintaining blood pressure either.

### 4.3. Uric Acid Was an Indicator for Cell Death

Uric acid was a metabolite from purines, while purines in the body came mainly from endogenously degraded nucleosides and partly from diet. Nucleosides might come from used DNA or RNA. A cell in a vigorous metabolism state needs many mRNAs to synthesize protein, and the used mRNAs will be degraded. Then, a part of them will be recycled to synthesize new mRNAs and a part will be transformed to uric acid. However, when a cell dies, the whole nucleus where abundant DNAs are located, along with the RNA in the cytoplasm, could be degraded, and many of which would be transformed to uric acid rather than recycled. Since the nucleus contains even more amount of nucleotides than the cytoplasm, a dead cell would result in more uric acid. By contrast with the yield of nucleosides from the nucleus, the yield of nucleosides from the cytoplasm meant nothing. So, there was no significant correlation between uric acid and energy metabolism. RCD is the main physiological way of cell death, and Casp3 is the main executor for RCD pathways [21]. Since uric acid in an organ was highly correlated to the expression level of the gene (Table 3), the results supported that uric acid was mainly resulted from dead cells. However, in a physiological state, dead cells are often replaced by regenerated cells, a process which is associated with Ki67 activation, an important nuclear protein [22, 23]. Results in Table 3 supported the deduction but with a smaller *R* value than Casp3. Since cell proliferation in the heart and liver was the main process in neonatal rats, there could be almost no cell death in the two neonatal organs (Figure 2). However, the intestinal tract both in neonatal rats and normal rats might be associated with many cell deaths and regeneration to remodel itself (Figures 1 and 2).

In the clinic, advanced cancer growth often couples with tumor cell death, and so does breast cancer. The degraded nucleosides in the dead tumor cells would be transformed into uric acid and caused an SUA increase (Figure 3(a)). Surprisingly, there was no significant correlation between Ki67 and SUA in patients with breast cancer but with a very slight tendency (*P* > 0.05, Figure S1). This phenomenon was different from that in normal rats. The reason might be associated with cell death inducing cell regeneration in normal rats, while tumor cell proliferation resulting in cell necrosis in patients with cancer. When breast cancer is treated with chemotherapeutics, many tumor cells would be killed. The nucleosides in the cells would be transformed into uric acid and caused an SUA increase (Figure 3(b)). Indeed, an SUA increase, including hyperuricemia and even gout, was widely regarded as an important marker for tumor lysis syndrome (TLS) [7, 28].

## 5. Conclusion

In summary, the dead cell was the main source for degraded nucleosides, and purines from the nucleosides would be transformed into uric acid locally. The uric acid would be transported to the blood and eventually would be excreted through the kidney or intestinal tract or deposited locally to cause tissue damage. The level of uric acid can be used as a reliable, though not sensitive sometimes, marker to evaluate cell death.

## Figures and Tables

**Table 1:** Top 20 pathways with significance could be associated with the level of uric acid in different organs.

ID	Description	Significant	Annotated	*P* value	*Q* value	Positive num.	Negative num.
ko03010	Ribosome	184/1937	288/7604	1.26E-44	3.37E-42	172	12
ko04141	Protein processing in the endoplasmic reticulum	89/1937	172/7604	8.09E-14	1.09E-11	62	27
ko00240	Pyrimidine metabolism	50/1937	106/7604	9.93E-07	8.89E-05	36	14
ko04975	Fat digestion and absorption	24/1937	39/7604	1.99E-06	0.000134	21	3
ko00520	Amino sugar and nucleotide sugar metabolism	27/1937	49/7604	8.96E-06	0.000481	19	8
ko05110	Vibrio cholerae infection	28/1937	52/7604	1.11E-05	0.000494	14	14
ko00230	Purine metabolism	68/1937	170/7604	1.88E-05	0.000721	38	30
ko00480	Glutathione metabolism	28/1937	54/7604	2.80E-05	0.000941	21	7
ko00983	Drug metabolism (other enzymes)	22/1937	39/7604	3.73E-05	0.001011	19	3
ko03460	Fanconi anemia pathway	27/1937	52/7604	3.77E-05	0.001011	18	9
ko03008	Ribosome biogenesis in eukaryotes	37/1937	81/7604	5.99E-05	0.001462	25	12
ko00620	Pyruvate metabolism	23/1937	43/7604	7.72E-05	0.001727	10	13
ko00330	Arginine and proline metabolism	28/1937	59/7604	0.000209	0.004278	17	11
ko00500	Starch and sucrose metabolism	21/1937	40/7604	0.000223	0.004278	12	9
ko03030	DNA replication	20/1937	38/7604	0.000301	0.00538	16	4
ko00900	Terpenoid backbone biosynthesis	14/1937	23/7604	0.000342	0.005742	10	4
ko00561	Glycerolipid metabolism	26/1937	56/7604	0.000534	0.008435	19	7
ko04972	Pancreatic secretion	40/1937	98/7604	0.000587	0.008748	33	7
ko04113	Meiosis (yeast)	26/1937	59/7604	0.001403	0.019819	19	7
ko04111	Cell cycle (yeast)	29/1937	70/7604	0.002443	0.032791	24	5
ko00970	Aminoacyl-tRNA biosynthesis	21/1937	47/7604	0.003207	0.03985	12	9
ko00510	N-Glycan biosynthesis	22/1937	50/7604	0.003266	0.03985	19	3
ko02020	Two-component system	10/1937	17/7604	0.003564	0.041593	6	4
ko04110	Cell cycle	45/1937	123/7604	0.003905	0.043679	42	3
ko04210	Apoptosis	33/1937	85/7604	0.004440	0.045337	21	12
ko03440	Homologous recombination	14/1937	28/7604	0.004476	0.045337	10	4
ko00100	Steroid biosynthesis	11/1937	20/7604	0.004560	0.045337	11	0
ko04910	Insulin signaling pathway	48/1937	134/7604	0.004738	0.045421	19	29
ko00564	Glycerophospholipid metabolism	35/1937	92/7604	0.005061	0.046841	23	12
ko03060	Protein export	12/1937	23/7604	0.005410	0.048403	8	4

**Table 2:** FPKM of genes mainly associated with urate metabolism and its correlation with the level of uric acid in different rat organs (mean ± SD, *n* = 3).

Gene	Brain	Kidney	Lung	Liver	Heart	Stomach	Duodenum	Terminal ileum	*R*	*P*
Xdh	2.57 ± 0.19	35.45 ± 3.79	66.10 ± 11.01	32.08 ± 1.73	26.76 ± 13.90	18.94 ± 5.88	281.88 ± 15.59	89.74 ± 14.30	0.9279	6.776E-11
Ada	1.62 ± 0.85	9.59 ± 2.00	19.59 ± 4.84	3.15 ± 0.61	7.83 ± 0.17	4.68 ± 1.10	1471.86 ± 703.35	225.33 ± 75.40	0.8874	3.79E-08
Uox	0.14 ± 0.15	0.03 ± 0.06	0.09 ± 0.08	384.58 ± 79.50	0 ± 0	0.08 ± 0.07	0.02 ± 0.03	0.084 ± 0.07	0.1128	0.5999

**Table 3:** FPKM of genes mainly associated with cell proliferation, apoptosis, and viability and its correlation with the level of uric acid in different rat organs (mean ± SD, *n* = 3).

Gene	Stomach	Kidney	Brain	Lung	Liver	Heart	Duodenum	Terminal ileum	*R*	*P*
Atpaf1	11.95 ± 3.43	13.11 ± 0.80	16.36 ± 2.17	6.48 ± 1.23	6.93 ± 1.99	15.12 ± 1.65	12.14 ± 2.04	10.72 ± 0.78	-0.3118	0.0972
Atpaf2	15.70 ± 1.12	16.74 ± 0.49	20.09 ± 0.87	10.76 ± 1.66	8.25 ± 0.59	23.01 ± 1.34	11.06 ± 2.69	11.14 ± 0.70	-0.6739	0.4541
Atp1a1	159.40 ± 49.43	1164.81 ± 7.36	448.52 ± 159.18	162.10 ± 12.58	126.8 ± 10.27	195.50 ± 69.00	930.40 ± 133.72	849.92 ± 95.97	0.3806	0.0665
Atp1a2	7.61 ± 2.98	4.78 ± 0.79	311.30 ± 57.23	14.52 ± 1.51	0.40 ± 0.41	63.22 ± 9.10	1.44 ± 0.23	7.37 ± 3.94	-0.4183	0.0419
Atp1a3	0.08 ± 0.06	0.10 ± 0.07	755.68 ± 95.52	3.25 ± 0.31	0.05 ± 0.07	1.48 ± 1.77	0.42 ± 0.15	0.48 ± 0.21	-0.3382	0.1060
Atp1a4	0 ± 0	0.92 ± 0.39	0.01 ± 0.01	0.05 ± 0.02	0 ± 0	0.04 ± 0.02	0 ± 0	0.04 ± 0.02	-0.2521	0.2347
Atp1b1	994.36 ± 127.15	2309.92 ± 166.22	1692.65 ± 175.76	101.26 ± 6.09	22.01 ± 6.89	307.94 ± 43.10	1345.89 ± 208.94	1265.77 ± 110.33	-0.0182	0.9329
Atp1b2	13.91 ± 2.54	6.28 ± 0.30	358.56 ± 211.59	3.79 ± 0.37	0.45 ± 0.02	12.90 ± 2.95	2.93 ± 0.68	4.27 ± 0.30	-0.3207	0.1265
Atp1b3	54.20 ± 5.53	63.09 ± 2.46	97.00 ± 16.34	169.85 ± 6.17	21.31 ± 1.16	48.63 ± 4.95	46.75 ± 4.69	40.10 ± 7.29	-0.2481	0.2425
Atp1b4	0 ± 0	0.01 ± 0.02	0 ± 0	1.10 ± 0.05	0 ± 0	2.73 ± 4.63	0.01 ± 0.01	0.01 ± 0.01	-0.2032	0.3410
Casp3	16.19 ± 4.95	4.73 ± 0.55	11.00 ± 1.25	15.79 ± 0.60	12.95 ± 1.30	4.96 ± 0.71	130.27 ± 27.41	41.27 ± 6.77	0.9045	1.325E-09
MKi67	5.29 ± 0.75	2.02 ± 0.41	0.50 ± 0.06	7.23 ± 2.60	1.74 ± 0.41	1.64 ± 1.11	12.99 ± 3.88	12.94 ± 2.19	0.7768	8.067E-06

## Data Availability

The data used to support the findings of this study are available from the corresponding author upon request.

## Supplementary Materials

Supplementary Materials Table S1: pathways with significance associated with the level of uric acid in different organs (*n* = 3). Figure S1: the relationship between SUA and Ki67 in patients with breast cancer (*n* = 203).

## References

[1] Cleophas M. C., Joosten L. A., Stamp L. K., Dalbeth N., Woodward O. M., Merriman T. R. (2017). ABCG2 polymorphisms in gout: insights into disease susceptibility and treatment approaches. *Pharmacogenomics and Personalized Medicine*.

[2] Yu K. H., Chen D. Y., Chen J. H. (2018). Management of gout and hyperuricemia: multidisciplinary consensus in Taiwan. *International Journal of Rheumatic Diseases*.

[3] Keebaugh A. C., Thomas J. W. (2010). The evolutionary fate of the genes encoding the purine catabolic enzymes in hominoids, birds, and reptiles. *Molecular Biology and Evolution*.

[4] Basseville A., Bates S. (2011). Gout, genetics and ABC transporters. *F1000 Biology Reports*.

[5] Maiuolo J., Oppedisano F., Gratteri S., Muscoli C., Mollace V. (2016). Regulation of uric acid metabolism and excretion. *International Journal of Cardiology*.

[6] Yun Y., Yin H., Gao Z. (2017). Intestinal tract is an important organ for lowering serum uric acid in rats. *PLoS One*.

[7] Cammalleri L., Malaguarnera M. (2007). Rasburicase represents a new tool for hyperuricemia in tumor lysis syndrome and in gout. *International Journal of Medical Sciences*.

[8] Hosomi A., Nakanishi T., Fujita T., Tamai I. (2012). Extra-renal elimination of uric acid via intestinal efflux transporter BCRP/ABCG2. *PLoS One*.

[9] Góth L. (2008). The rasburicase therapy may cause hydrogen peroxide shock. *Orvosi Hetilap*.

[10] Alvarez-Lario B., Macarron-Vicente J. (2010). Uric acid and evolution. *Rheumatology*.

[11] Johnson R. J., Titte S., Cade J. R., Rideout B. A., Oliver W. J. (2005). Uric acid, evolution and primitive cultures. *Seminars in Nephrology*.

[12] Tian H., Ye X., Hou X., Yang X., Yang J., Wu C. (2016). SVCT2, a potential therapeutic target, protects against oxidative stress during ethanol-induced neurotoxicity *via* JNK/p38 MAPKs, NF-*κ*B and miRNA125a-5p. *Free Radical Biology and Medicine*.

[13] Zakharova I., Sokolova T., Vlasova Y., Bayunova L., Rychkova M., Avrova N. (2017). *α*-Tocopherol at nanomolar concentration protects cortical neurons against oxidative stress. *International Journal of Molecular Sciences*.

[14] Carito V., Ceccanti M., Tarani L., Ferraguti G., Chaldakov G. N., Fiore M. (2016). Neurotrophins’ modulation by olive polyphenols. *Current Medicinal Chemistry*.

[15] Yin H., Hou X., Tao T., Lv X., Zhang L., Duan W. (2015). Neurite outgrowth resistance to rho kinase inhibitors in PC12 Adh cell. *Cell Biology International*.

[16] Chen H., Cao G., Chen D. Q. (2016). Metabolomics insights into activated redox signaling and lipid metabolism dysfunction in chronic kidney disease progression. *Redox Biology*.

[17] Trapnell C., Williams B. A., Pertea G. (2010). Transcript assembly and quantification by RNA-Seq reveals unannotated transcripts and isoform switching during cell differentiation. *Nature Biotechnology*.

[18] Lin Y., Zhu J., Wang Y., Li Q., Lin S. (2017). Identification of differentially expressed genes through RNA sequencing in goats (*Capra hircus*) at different postnatal stages. *PLoS One*.

[19] Mohamedali K. A., Guicherit O. M., Kellems R. E., Rudolph F. B. (1993). The highest levels of purine catabolic enzymes in mice are present in the proximal small intestine. *Journal of Biological Chemistry*.

[20] Snigdha S., Smith E. D., Prieto G. A., Cotman C. W. (2012). Caspase-3 activation as a bifurcation point between plasticity and cell death. *Neuroscience Bulletin*.

[21] Galluzzi L., Vitale I., Aaronson S. A. (2018). Molecular mechanisms of cell death: recommendations of the Nomenclature Committee on Cell Death 2018. *Cell Death & Differentiation*.

[22] Zhou Y., Hu W., Chen P. (2017). Ki67 is a biological marker of malignant risk of gastrointestinal stromal tumors: a systematic review and meta-analysis. *Medicine*.

[23] Gerring Z., Pearson J. F., Morrin H. R., Robinson B. A., Harris G. C., Walker L. C. (2015). Phosphohistone H3 outperforms Ki67 as a marker of outcome for breast cancer patients. *Histopathology*.

[24] Watanabe S., Kang D. H., Feng L. (2002). Uric acid, hominoid evolution, and the pathogenesis of salt-sensitivity. *Hypertension*.

[25] Hyndman D., Liu S., Miner J. N. (2016). Urate handling in the human body. *Current Rheumatology Reports*.

[26] Gutman A. B. (1965). Significance of uric acid as a nitrogenous waste in vertebrate evolution. *Arthritis & Rheumatism*.

[27] Mortada I. (2017). Hyperuricemia, type 2 diabetes mellitus, and hypertension: an emerging association. *Current Hypertension Reports*.

[28] Koratala A. (2017). Tumor lysis syndrome with massive hyperphosphatemia and hyperuricemia. *Clinical Case Reports*.

