# Supplementary material for: What Is the Biological Function of Uric Acid? An Antioxidant for Neural Protection or a Biomarker for Cell Death
Source: Dis Markers. 2019 Jan 10;2019:4081962. doi: 10.1155/2019/4081962 (PMC6348815; doi:10.1155/2019/4081962)
Supplement: Supplementary Materials — Table S1: pathways with significance associated with the level of uric acid in different organs (n = 3). Figure S1: the relationship between SUA and Ki67 in patients with breast cancer (n = 203). [file 4081962.f1.doc]

S1-Table Pathways with significance associated with the level of uric acid in different organs (n=3)

| ID | Description | Significant | Annotated | Pvalue | Qvalue | Positive Num | Negative Num |
| --- | --- | --- | --- | --- | --- | --- | --- |
| ko03010 | Ribosome | 184/1937 | 288/7604 | 1.26E-44 | 3.37E-42 | 172 | 12 |
| ko04141 | Protein processing in endoplasmic reticulum | 89/1937 | 172/7604 | 8.09E-14 | 1.09E-11 | 62 | 27 |
| ko00240 | Pyrimidine metabolism | 50/1937 | 106/7604 | 9.93E-07 | 8.89E-05 | 36 | 14 |
| ko04975 | Fat digestion and absorption | 24/1937 | 39/7604 | 1.99E-06 | 0.000134 | 21 | 3 |
| ko00520 | Amino sugar and nucleotide sugar metabolism | 27/1937 | 49/7604 | 8.96E-06 | 0.000481 | 19 | 8 |
| ko05110 | Vibrio cholerae infection | 28/1937 | 52/7604 | 1.11E-05 | 0.000494 | 14 | 14 |
| ko00230 | Purine metabolism | 68/1937 | 170/7604 | 1.88E-05 | 0.000721 | 38 | 30 |
| ko00480 | Glutathione metabolism | 28/1937 | 54/7604 | 2.80E-05 | 0.000941 | 21 | 7 |
| ko00983 | Drug metabolism - other enzymes | 22/1937 | 39/7604 | 3.73E-05 | 0.001011 | 19 | 3 |
| ko03460 | Fanconi anemia pathway | 27/1937 | 52/7604 | 3.77E-05 | 0.001011 | 18 | 9 |
| ko03008 | Ribosome biogenesis in eukaryotes | 37/1937 | 81/7604 | 5.99E-05 | 0.001462 | 25 | 12 |
| ko00620 | Pyruvate metabolism | 23/1937 | 43/7604 | 7.72E-05 | 0.001727 | 10 | 13 |
| ko00330 | Arginine and proline metabolism | 28/1937 | 59/7604 | 0.000209 | 0.004278 | 17 | 11 |
| ko00500 | Starch and sucrose metabolism | 21/1937 | 40/7604 | 0.000223 | 0.004278 | 12 | 9 |
| ko03030 | DNA replication | 20/1937 | 38/7604 | 0.000301 | 0.00538 | 16 | 4 |
| ko00900 | Terpenoid backbone biosynthesis | 14/1937 | 23/7604 | 0.000342 | 0.005742 | 10 | 4 |
| ko00561 | Glycerolipid metabolism | 26/1937 | 56/7604 | 0.000534 | 0.008435 | 19 | 7 |
| ko04972 | Pancreatic secretion | 40/1937 | 98/7604 | 0.000587 | 0.008748 | 33 | 7 |
| ko04113 | Meiosis - yeast | 26/1937 | 59/7604 | 0.001403 | 0.019819 | 19 | 7 |
| ko04111 | Cell cycle - yeast | 29/1937 | 70/7604 | 0.002443 | 0.032791 | 24 | 5 |
| ko00970 | Aminoacyl-tRNA biosynthesis | 21/1937 | 47/7604 | 0.003207 | 0.03985 | 12 | 9 |
| ko00510 | N-Glycan biosynthesis | 22/1937 | 50/7604 | 0.003266 | 0.03985 | 19 | 3 |
| ko02020 | Two-component system | 10/1937 | 17/7604 | 0.003564 | 0.041593 | 6 | 4 |
| ko04110 | Cell cycle | 45/1937 | 123/7604 | 0.003905 | 0.043679 | 42 | 3 |
| ko04210 | Apoptosis | 33/1937 | 85/7604 | 0.00444 | 0.045337 | 21 | 12 |
| ko03440 | Homologous recombination | 14/1937 | 28/7604 | 0.004476 | 0.045337 | 10 | 4 |
| ko00100 | Steroid biosynthesis | 11/1937 | 20/7604 | 0.00456 | 0.045337 | 11 | 0 |
| ko04910 | Insulin signaling pathway | 48/1937 | 134/7604 | 0.004738 | 0.045421 | 19 | 29 |
| ko00564 | Glycerophospholipid metabolism | 35/1937 | 92/7604 | 0.005061 | 0.046841 | 23 | 12 |
| ko03060 | Protein export | 12/1937 | 23/7604 | 0.00541 | 0.048403 | 8 | 4 |
| ko00601 | Glycosphingolipid biosynthesis - lacto and neolacto series | 14/1937 | 29/7604 | 0.006653 | 0.057607 | 10 | 4 |
| ko00030 | Pentose phosphate pathway | 14/1937 | 30/7604 | 0.009598 | 0.078646 | 10 | 4 |
| ko00051 | Fructose and mannose metabolism | 15/1937 | 33/7604 | 0.009962 | 0.078646 | 10 | 5 |
| ko00710 | Carbon fixation in photosynthetic organisms | 15/1937 | 33/7604 | 0.009962 | 0.078646 | 10 | 5 |
| ko05134 | Legionellosis | 24/1937 | 61/7604 | 0.011708 | 0.089794 | 20 | 4 |
| ko00052 | Galactose metabolism | 14/1937 | 31/7604 | 0.013475 | 0.097414 | 7 | 7 |
| ko03410 | Base excision repair | 18/1937 | 43/7604 | 0.013613 | 0.097414 | 13 | 5 |
| ko00513 | Various types of N-glycan biosynthesis | 16/1937 | 37/7604 | 0.013791 | 0.097414 | 13 | 3 |
| ko04120 | Ubiquitin mediated proteolysis | 49/1937 | 145/7604 | 0.014823 | 0.102024 | 20 | 29 |
| ko04974 | Protein digestion and absorption | 30/1937 | 82/7604 | 0.016502 | 0.110739 | 27 | 3 |
| ko03040 | Spliceosome | 48/1937 | 144/7604 | 0.020404 | 0.133579 | 39 | 9 |
| ko04612 | Antigen processing and presentation | 29/1937 | 81/7604 | 0.024668 | 0.155194 | 25 | 4 |
| ko00760 | Nicotinate and nicotinamide metabolism | 13/1937 | 30/7604 | 0.024862 | 0.155194 | 9 | 4 |
| ko00600 | Sphingolipid metabolism | 19/1937 | 49/7604 | 0.027446 | 0.166467 | 14 | 5 |
| ko03013 | RNA transport | 55/1937 | 171/7604 | 0.028146 | 0.166467 | 43 | 12 |
| ko04530 | Tight junction | 44/1937 | 133/7604 | 0.029071 | 0.166467 | 27 | 17 |
| ko04114 | Oocyte meiosis | 37/1937 | 109/7604 | 0.029148 | 0.166467 | 22 | 15 |
| ko00533 | Glycosaminoglycan biosynthesis - keratan sulfate | 8/1937 | 16/7604 | 0.03009 | 0.168267 | 3 | 5 |
| ko04930 | Type II diabetes mellitus | 19/1937 | 50/7604 | 0.033992 | 0.186209 | 9 | 10 |
| ko03420 | Nucleotide excision repair | 18/1937 | 47/7604 | 0.035555 | 0.190875 | 11 | 7 |
| ko04978 | Mineral absorption | 17/1937 | 44/7604 | 0.037132 | 0.194452 | 16 | 1 |
| ko01230 | Biosynthesis of amino acids | 30/1937 | 87/7604 | 0.03767 | 0.194452 | 19 | 11 |
| ko00440 | Phosphonate and phosphinate metabolism | 4/1937 | 6/7604 | 0.040087 | 0.203024 | 4 | 0 |
| ko00450 | Selenocompound metabolism | 8/1937 | 17/7604 | 0.044433 | 0.220868 | 5 | 3 |
| ko04115 | p53 signaling pathway | 24/1937 | 68/7604 | 0.04542 | 0.221667 | 23 | 1 |
| ko04142 | Lysosome | 39/1937 | 120/7604 | 0.049551 | 0.235631 | 11 | 28 |

S2-Figure The relationship between serum uric acid and Ki67 in patients with breast cancer (n=203). There was no significant correlation between them (P>0.05).
